# Reduction in all-cause medical and caregiving costs through innovative health awareness projects in a rural area in Japan: a retrospective cohort study

**DOI:** 10.1186/s12913-024-10836-0

**Published:** 2024-03-25

**Authors:** Ayako Shoji, Kennichi Kudo, Koichi Murashita, Shigeyuki Nakaji, Ataru Igarashi

**Affiliations:** 1 Department of Health Economics and Outcomes Research, Graduate School of Pharmaceutical Sciences, The University of Tokyo, 7-3-1, Hongo, Bunkyo-Ku, Tokyo, 113-0033 Japan; 2 Healthcare Consulting, Inc, 1-8-19, Fujimi, Chiyoda-Ku, Tokyo, 102-0071 Japan; 3 Research Institute of Health Innovation, Hirosaki University, 5 Zaifu-Cho, Hirosaki City, Aomori 036-8562 Japan; 4 Department of Social Medicine, Graduate School of Medicine, Hirosaki University, 5 Zaifu-Cho, Hirosaki City, Aomori 036-8562 Japan; 5 Department of Public Health and Preventive Medicine, School of Medicine, Yokohama City University, 3-9 Fukuura, Kanazawa-Ku, Yokohama City, Kanagawa 236-0004 Japan

**Keywords:** Health program, Rural area, Medical costs, Caregiving costs, Data linkage

## Abstract

**Background:**

This study evaluates cost reduction in participants of a health awareness program (the Center of Healthy Aging Program, CHAP) in a Japanese rural area, characterized by an annual check-up and personalized interview on health issues and related risks immediately after the check-up.

**Methods:**

This is a cross-sectional study using medical and caregiving costs and Japan-specific health check-up results in Hirosaki residents stored by the local government, which were individually-based linked to the CHAP information collected by Hirosaki University. This is the first study that used anonymized data with individually-based linkages to both a research institute and a local government in Japan under a strict limitation regarding linking to third-party data. We included residents who had been continuously enrolled for > 6 months as of 1 July 2015. We compared 5-year all-cause costs between three groups (with CHAP, with Japan-specific health check-up, and no check-up) using a multivariate negative binomial regression model considering risk factors including lifestyle habits and an inverse probability weight to adjust for baseline characteristics: age, sex, Charlson comorbidity index, baseline care level, and risk score of coronary heart diseases.

**Results:**

A total of 384, 9805, and 32,630 residents aged 40–74 years were included for the CHAP, Japan-specific health check-up, and no check-up groups, respectively. The Japan-specific health check-up group showed older and higher Charlson comorbidity index than the others. After inverse probability weight adjusting, the amount of all-cause medical costs was significantly lower only in the CHAP group. Faster walking speed and exercise habits were independently associated with lower all-cause medical and caregiving costs.

**Conclusions:**

We demonstrated a 5-year all-cause cost reduction in residents who participated in the CHAP and also suggested the effect of exercise habits in Hirosaki, which indicated the significance of individually-based data linkages to external third-party data for all local governments to improve the health condition of residents.

**Supplementary Information:**

The online version contains supplementary material available at 10.1186/s12913-024-10836-0.

## Introduction

Health conditions vary among communities, and are associated with differences in income, health behaviours, supplies of healthcare resources, education levels, local government expenditures, and local area characteristics [1–3]. Furthermore, rural communities sometimes face more serious health conditions leading to high mortality rates owing to older and/or low-income residents [4–6], which may contribute to both an increase in the healthcare burden and financial stress. The weaker financial base in smaller local governments hinders their ability to implement long-term community-based measures.

**Fig. 1:**
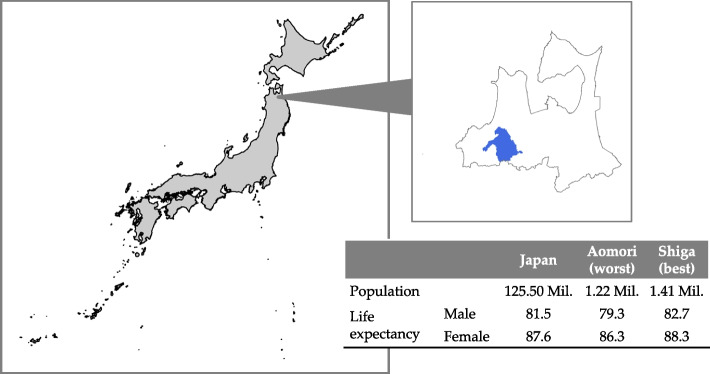
The location of Aomori Prefecture and Hirosaki City

Japan is a rapidly aging country with the highest proportion of elderly people in the world. According to the Ministry of Internal Affairs and Communications, in 2021, people aged 65 and over accounted for 28.9% of the total population [7]. The disparity in average lifetime between different areas in Japan is 3.5 years in men or 2.0 years in women (Fig. 1). To prevent premature death, a national health check-up (Japan-specific health check-up, SHC) and health guidance program are provided to the whole population aged 40 years and over. Although the program has been shown to be beneficial [8–10], the participation rate varied from 25.5% to 47.2% among areas and its long-term benefits are controversial [10]. The Aomori Prefecture, a typical rural area located in northern Japan (Fig. 1), has the shortest life expectancy since records began, and the differences from the national average and from the longest value have expanded over the last 20 years. Hirosaki University (Aomori, Japan) started an awareness and encouragement program—the Center of Healthy Aging Program (CHAP) in 2013 in Hirosaki [11], the third-largest city in the Aomori Prefecture, to improve overall life quality and longevity. The CHAP combines an annual check-up and personalized interview, where participants are informed about health issues and related risks based on individual results immediately after the check-up.

We have demonstrated the decreased risks of coronary heart diseases (CHDs) and stroke after the start of CHAP. Furthermore, assuming the participation of all residents, which amounted to 1.29 million in the Aomori Prefecture [11], we estimated that the CHAP would lead to a reduction of 22,486 and 9,603 patients and a reduction in JPY 21,973 (USD 156.01 or EUR 138.43, JPY 100 = USD 0.71 or EUR 0.63) and JPY 16,056 million (USD 114.00 or EUR 101.15) in medical costs over a 10-year period by avoiding CHDs and stroke, respectively. However, in the study, we extrapolated medical costs for CHD and stroke per patient from the aggregated data of the entire Aomori Prefecture presented in the latest governmental statistics, which does not reflect the area-specific health conditions. Individual data on health resource utilization for CHAP participants are owned by the Hirosaki city government, but in Japan, the sharing of data that enables individually-based linking to data owned by third parties is strictly limited. To the best of our knowledge, the present study was the first to utilize anonymized data with linkage from both a research institute and a local government in Japan. The study aimed to compare all-cause medical and caregiving cost reduction using individual data on resource utilisation between participants and non-participants of the CHAP.

## Methods

This was a cross-sectional study in which Hirosaki University, Hirosaki city, and the University of Tokyo collaborated to confirm the impact of an innovative health promotion program on the residents’ health. The study was conducted in accordance with the Declaration of Helsinki from 1964 and its later amendments, and with the approval of the Ethics Committee of the University of Tokyo Faculty of Medicine (2023080NI).

### Study population

We included residents who were enrolled in the residence-based insurance administered by the government of Hirosaki City in Aomori Prefecture, located in the northern part of Japan, were included in this study (Fig. 1). Hirosaki is associated with unhealthy lifestyle factors such as a high rate of smoking, high percentage of obesity, high alcohol consumption, high salt intake, and low vegetable intake than other prefectures [12]. These included those who were unemployed or self-employed residents and their non-working dependents. All participants had been continuously enrolled for more than 6 months as of 1 July 2015 (index date). In Japan, insurance comprises over 3000 providers and can be classified into three types: employee-based health insurance, residence-based insurance, and health insurance for individuals in the late-stage elderly category (i.e., those 75 years and over) [13]. Approximately half of the residents in Hirosaki City are involved in the agriculture, forestry, or fishery industry, and as a result, the enrolment of the residence-based insurance is anticipated to closely reflect the population within this sector. Residents who had withdrawn from the insurance at the index date or who chose to opt-out were excluded.

We classified the included residents into three groups based on their history and type of check-ups during the previous year: CHAP, Japan-specific health check-up (SHC), and no check-up. The CHAP is provided to residents living in the Iwaki area, accounting for approximately 6% of the total number of residents in Hirosaki City. Developed to increase the health awareness of residents, the CHAP combines an annual check-up with more than 3,000 check-up items and a personalised interview in which participants are informed of their health issues and related risks based on individual results. In contrast, the SHC is a national program provided by the government of Hirosaki City. It targets insured residents aged 40–74 years and is mandatory for all insurance providers, including residence-based insurance societies. It is conducted annually using common check-up items (e.g. body composition, blood pressure, blood test results, and lifestyle habits) [14, 15]. Unlike the CHAP, the SHC does not provide any further information, except to high-risk participants, about health issues and risks, nor does it recommend any particular lifestyle. The no check-up group included residents who had been recommended to receive the SHC but had not responded or could not receive it due to illness and those who were ineligible for the SHC (aged under 40 years or 75 years and over). Therefore, to maintain comparability, we included participants aged 40–74 years in our analyses.

### Data sources

We used anonymized resident information and medical and long-term care insurance claims data of the included residents, as well as the results of the SHC for 5 years from the index date, which were provided to Hirosaki University by the government of Hirosaki City, and were individually linked to the Iwaki Cohort data and anonymized. The cohort data have been collected by Hirosaki University since 2005 [12] and contain results of check-ups as part of the CHAP. All participants in the cohort provided written informed consent to Hirosaki University [12]. The resident information contains the enrolment date and the date on which residents lost their insurance coverage. The medical claims data contain sex, birth year and month, disease names and the dates when healthcare resources were utilized, and the information on utilized resources such as drugs, procedures, and materials, including their amount and price. The care claims data contain the type of care services and corresponding costs and dates. Informed consent was obtained by allowing all residents of Hirosaki City to opt-out of the study through an official city newsletter. We had no access to information that could identify individual participants during or after data collection.

Some participants in the CHAP group also received the SHC check-up; thus, they had two types of results. For participants with both results, we only used the results of the SHC.

### Endpoints

The primary endpoint was all-cause medical costs during the observation period up to 5 years after the index date. As the CHAP did not focus on the prevention of specific diseases but aimed at total health promotion, we included all-cause medical costs. These were compared between the three groups and limited to participants aged 40 to 74 years at the index date because the SHC is provided only for this age group. The observation period ended for participants who were withdrawn from the insurance. To adjust for risk factors, the Charlson Comorbidity Index (CCI)[16] was calculated for every participant based on the diagnosis without any suspicious flags (registered by physicians if they had insufficient information to provide a definitive diagnosis) during the 6 months preceding the index date (i.e., 1 January 2015 to 30 June 2015). This study included relatively healthy people without any disorders; however, we used the CCI as a risk factor because residents in the no-check-up group had no data reflecting health conditions other than claims data. Participants without a diagnosis corresponding to the CCI during that period were considered to not have any high-risk disease (i.e., CCI of 0). The risk score of CHDs, which was developed based on a population-based prospective cohort study in Japan and predicts the 10-year probability of developing CHD [17] (Table S1 and S2 in Supporting Material 1) was not adapted for the primary endpoint, because it requires blood pressure and blood test results, both of which the no exam group did not have, although it is also considered a risk factor for future medical costs.

The secondary endpoints were CHD-related medical costs and all-cause total costs, including not only medical costs but also caregiving costs. For the former, we defined the costs of participants diagnosed with CHDs (ICD-10 codes I20.x, I21.x, I22.x, I23.x, I24.x, and I25.x) as CHD-related costs. For the latter, we aimed to confirm the effect of improving lifestyle habits on caregiving resource utilization. This is because the 5-year period is considered too short to prevent lifestyle diseases but has potential in preventing fragility. The caregiving costs were expected to be higher in residents with low motor function, which was identified only in the SHC group and the CHAP group as exercise habits (engaging in exercising that induces sweating two or more times a week for more than 30 min) (yes or no) and walking speed compared with a person of the same generation and the same sex (faster or not) [15]. Therefore, in the analysis, we excluded the no check-up group and considered the risk score of CHDs as an adjusting variable, which we were able to calculate using the check-up results.

### Statistical analysis

We used the hurdle negative binomial regression model, because only a subset of participants who experience the onset of any disease needs healthcare resources. We considered the log of the individual exposure duration as an offset variable to account for the varying lengths of the observable periods among the participants. We confirmed the goodness of fit of the model compared with the hurdle Poisson regression model and generalised negative binomial model using Akaike’s Information Criterion (AIC).

To adjust for the differences in characteristics between the three groups, additional weighted analyses were performed using the inverse probability weight, which was obtained from a multi-logit regression model based on all variables considered: age, sex, CCI, baseline care level in the primary analysis, and age, sex, CCI, baseline care level, risk score of CHDs, exercise habits and walking speed in the secondary analysis. Standardized differences of less than 0.1 and the lowest Rubin index value were the criteria used for the best balance of baseline. The care level was evaluated for residents who found it difficult to live by themselves and who needed care services from the local government.

In the secondary analysis for all-cause total costs, the association between lifetime habits and costs for 5 years after the index date was also confirmed. However, data on exercise habits and walking speed were missing for participants who had been examined but did not answer the questionnaire about lifetime habits. Multiple imputation was applied to the pooled dataset to generate 10 imputed datasets via a cart using all-cause total costs, age, sex, CCI score, risk score, baseline care level, exercise habits, and walking speed. The analysis was performed across all imputed datasets using Rubin’s rules to obtain a set of final estimates.

All statistical analyses were carried out using R software, version 4.1.2 (R Project for Statistical Computing) and the following R packages: twang, WeightIt, mice, and MatchThem. All statistical tests were two tailed, and a significance level of 5% was used.

## Results

### Patients’ characteristics

**Fig. 2:**
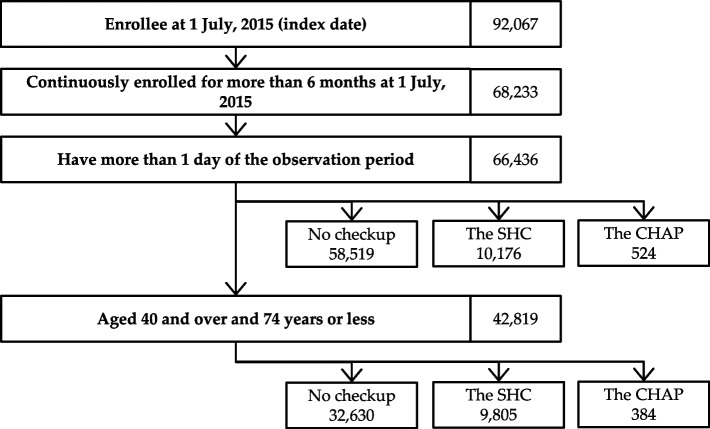
Flow of identifying subjects of analyses

Of the 68,233 enrolees at the index date, 66,436 were eligible, which covered about 37% of the entire population in Hirosaki City on 1 July 2015. After excluding residents aged less than 40 years or 75 years and over, 384, 9805, and 32,630 were included in the analysis for the CHAP, SHC, and no check-up groups, respectively (Fig. 2). Participants in the SHC group were older and had higher CCI both before and after excluding the population aged less than 40 years or 75 years and over. The baseline care level was lowest in the CHAP group (Table 1). The all-cause medical costs per person per observation year was JPY 651,426 (USD 4,625.13 or EUR 4,103.98). Table S3 shows the all-cause medical costs per person-year by subcategory and by year of follow-up. Fig. S1 shows this distribution.

**Table 1:** Characteristics of the enrolled residents

		No check-up			SHC			CHAP		
		n, mean	%, sd	missing	n, mean	%, sd	missing	n, mean	%, sd	missing
N		32630			9805			384		
Age (yrs) (mean, sd)		60.50	9.70	0	64.77	7.98	0	61.43	8.63	0
Sex (women) (n, %)		16657	51.05	0	5706	58.19	0	233	60.68	0
CCI (mean, sd)		0.74	1.60	0	0.89	1.42	0	0.67	1.07	0
Care level (n, %)	Level 0 (no need of care)	32238	98.80	0	9748	99.42	0	383	99.74	0
	Level 1	40	0.12	0	22	0.22	0	0	0.00	0
	Level 2	38	0.12	0	9	0.09	0	1	0.26	0
	Level 3	76	0.23	0	10	0.10	0	0	0.00	0
	Level 4	111	0.34	0	12	0.12	0	0	0.00	0
	Level 5	56	0.17	0	2	0.02	0	0	0.00	0
	Level 6	36	0.11	0	0	0.00	0	0	0.00	0
	Level 7	35	0.11	0	2	0.02	0	0	0.00	0
Risk score (CHDs) (mean, sd)		−	−	32630	39.09	9.00	0	36.90	8.79	0
Exercise habits (n, %)^a^		−	−	32630	1001	33.90	6852	72	22.15	59
Walking speed^b^ (n, %)		−	−	32630	1271	43.03	6851	135	41.54	59

*CCI* Charlson Comorbidity Index, *CHD* Coronary heart disease, *SHC* Standard health check-up, *yrs* Years

^a^Exercise habits mean engaging in exercise that induces sweating two or more times a week for more than 30 min (yes or no)

^b^Walking speed compared with a person of the same generation and sex (faster or slower)

### Primary analysis

The incidence of all-cause medical costs was significantly higher in both the SHC and CHAP groups, and the amount of all-cause medical costs was significantly lower in both SHC and CHAP group compared with the no check-up group. The result was similar in the IPW-adjusted analysis but the amount of all-cause medical costs was significantly lower only in CHAP group (Table 2). CCI was associated with a significantly higher incidence rate and amount of all-cause medical costs. According to the estimated parameters, the cost reductions attributed to the CHAP amounted to JPY 302684 (USD 2149.06 or EUR 1906.91) per person, assuming males aged 60 years with a CCI of one (one high-risk disease or disease group), while no significant cost reduction was observed for the SHC. The proportion of patients without any all-cause medical expenditure was 17.6% among the analysed residents, which supported the use of the hurdle or negative binomial regression model. The AICs for the applied models were the smallest for the negative binomial regression model (11,158.8), followed by the generalised negative binomial model (11,423.73) and the Poisson regression model (1,145,567,338).

**Table 2:** Difference in the amount and incidence ofall-cause medical costs among groups^a^

		Count				Incidence			
		Estimated	RR	95% CI	*P*-value	Estimated	OR	95% CI	*P*-value
Check-up type	No check-up	Reference				Reference			
	SHC	− 0.028	0.972	(0.700–1.350)	0.865	2.269	9.671	(3.265–28.651)	< 0.001
	CHAP	− 0.438	0.645	(0.458–0.910)	0.013	1.269	3.556	(1.565–8.082)	0.002

*OR* Odds ratio, *RR* Risk score, *SHC* Standard health check-up, *yrs* Years

^a^The covariates used to adjust for differences in characteristics between the groups were age, sex, and the Charlson Comorbidity Index (CCI)

### Secondary analysis

The incidence of CHD-related medical costs was significantly higher and the amount of CHD-related medical costs was significantly lower in both SHC and CHAP groups compared with the no check-up group. However, after IPW-adjusting, only the CHAP group was associated with a higher incidence and lower amount of CHD-related medical costs (Table 3).

**Table 3:** Difference in the amount and incidence of CHD-related costs among groups^a^

		Count				Incidence			
		Estimated	RR	95% CI	*P*-value	Estimated	OR	95% CI	*P*-value
Check-up type	No check-up	Reference				Reference			
	SHC	− 0.212	0.809	(0.505–1.296)	0.378	0.257	1.292	(0.727–2.298)	0.382
	CHAP	− 0.503	0.605	(0.377–0.970)	0.037	0.586	1.797	(1.004–3.218)	0.048

*CHD* coronary heart disease, *OR* odds ratio, *RR* risk score, *SHC* standard health check-up, *yrs* Years

^a^The covariates used to adjust for differences in characteristics between the groups were age, sex, and the Charlson Comorbidity Index (CCI)

Both incidence and amount of all-cause total costs were significantly lower in the CHAP group compared with the SHC group in the IPW-adjusted analysis. Faster walking speed and exercise habits were associated with a lower amount of costs. After the missing data were imputed, the results were similar (Table 4,6). The density plots of the original and imputed datasets are shown in Fig. S2 in Supporting Material. On the other hand, it might associate with any bias due to the characteristics of missing values. Assuming males aged 60 years with CCI of one (one comorbidity) and CHD risk score of 35, the cost reductions owing to having exercise habits and higher walking speed were JPY 103830 (USD 737.20 or EUR 654.13) and JPY 230632 (USD 1637.49 or EUR 1452.98) per person, respectively. The CHAP independently contributed to a reduction of JPY 322981 (USD 2293.17 or EUR 2034.78) per person.

**Table 4:** Difference in the amount and incidence of all-cause medical and caregiving costs among groups^a^

		Count				Incidence			
		Estimated	RR	95% CI	*P*-value	Estimated	OR	95% CI	*P*-value
**Before imputing**									
Check-up type	SHC	Reference				Reference			
	CHAP	− 0.171	0.843	(0.799–0.890)	< 0.001	0.712	2.038	(1.315–3.158)	0.001
**After imputing**									
Check-up type	SHC	Reference				Reference			
	CHAP	− 0.425	0.654	(0.576–0.742)	< 0.001	− 1.211	0.298	(0.197–0.450)	< 0.001

*OR* odds ratio, *RR* Risk score, *SHC* Standard health check-up, *yrs* Years

^a^The covariates used to adjust for differences in characteristics between the groups were age, sex, risk score (coronary heart disease), baseline care level, exercise habits, walking speed, and the Charlson Comorbidity Index (CCI). Exercise habits refer to engaging in exercise that induces sweating two or more times a week for more than 30 min (yes or no). Walking speed compared with a person of the same generation and sex (faster or slower)

## Discussion

We revealed that the CHAP, an innovative awareness program, reduced all-cause medical and caregiving costs within a 5-year period. These findings were based on the actual healthcare resource utilization data of individual residents. Our findings are significant as they stem from the first-ever linkage study utilizing individual data owned by a local government and a university in Japan. In a previous study [11], we demonstrated the cost reduction effect of the CHAP using the aggregated total medical costs in the Aomori Prefecture, but did not consider individual variation caused by comorbidities and health behaviours. In this study, we confirmed that individual costs were reduced in the CHAP group, after adjusting for differences in demographic and clinical characteristics.

Generally, health awareness programs aim to encourage high-risk individuals to visit physicians [18–20] or to change their habits and attitudes [21–23]. Both of these strategies are expected to result in a long-term health maintenance and healthcare cost reduction, for which the 5-year evaluation period is short. Actually, in the SHC groups, CHD-related costs were similar to those in the no check-up group in the IPW-adjusted analyses. However, there was a significant reduction in the analysis without adjusting, suggesting that the difference in 5-year CHD-related costs was strongly associated with the difference in baseline conditions between the groups. Basically, residents who receive any check-ups are considered to have low care levels or enough health consciousness to participate in health programs [24]. Higher CCIs in the SHC group might suggest that the comorbidity of chronic diseases encourages individuals to participate in routine check-ups [25]. Nonetheless, the finding that the CHAP remained associated with a significant reduction in all-cause costs in the IPW-adjusted analyses indicates an additional benefit of the CHAP in preventing expensive therapies independently of observed baseline conditions.

In the present study, participants in both programs were more likely to incur costs, specifically in terms of more frequent visits to physicians compared with non-participants, because both programs recommend participants with abnormal check-up results to have detailed examinations in clinics. The noteworthy characteristics of the check-up as part of the CHAP compared with the SHC is that it explains the results immediately after the check-up. This provides individuals with a deep understanding of their health followed by a behaviour modification and an early initiation of treatment to prevent the onset of diseases accompanied by more expensive treatments. These characteristics promote outpatient visits over the next year but possibly reduce them in the succeeding years through the improvement in health conditions. Conversely, the results of the SHC are less detailed. Such difference is believed to account for the disparity in the reduction of both the incidence and amount of all-cause costs between the CHAP and SHC groups. The all-cause medical costs per person-year by subcategory (i.e. inpatient, outpatient, and medication) and by year of follow-up showed that the inpatient costs in the CHAP group remained at a relatively lower level than those in the no check-up group compared to other cost categories (Table S2).

Public medical insurance providers, including local governments, who provide enrolees with the opportunity to opt-out (passive consent), are able to utilize anonymized data without data linkage in collaboration with academic and research organizations, solely for getting valuable information to maintain and improve enrolees’ wellness [26]. Under this process, the limited long-term effect of a local government health program on medical cost reduction was reported [26]. The nationwide SHC program has been required to be offered by law since 2008, but a recent report indicated that it had no effect on improving or maintaining laboratory values [10].

We used anonymized data owned by a local government and a university with data linkage and observed a significant effect of CHAP on healthcare cost reductions. To develop effective health programs beyond standardized approaches, analyses using individually-based linked data to external data owned by third parties who have innovative health programs should be required by local governments. We believe this result can be useful in setting goals to encourage residents. In programs that promote health behaviours, local governments frequently provide laboratory values as goals, such as blood pressure, blood glucose, cholesterol, or risk scores of CHDs and stroke, and leave residents to decide how to modify their daily habits to achieve the goals. While such programs might improve health conditions in residents with high health literacy, residents with low health literacy are often stuck behind. Clear behavioural goals, such as implementing a habit of exercising twice per week or more than 30-min or faster walking are accessible and easy to start, irrespective of individuals’ living environment or health literacy.

### Limitations of this study

Several limitations should be mentioned. First, we classified eligible residents into three groups solely based on the history and type of check-ups during the year before the index date. Therefore, there was no differentiation between residents who annually received check-ups and those who incidentally received them, even though there may be a difference regarding all-cause medical and caregiving costs between them. Second, our results may not fully reflect the effects of the programs themselves. This is because of the possible presence of unmeasured confounders, which could not be adequately controlled owing to limited information. Third, residents aged 75 years and over were not included in the study, although they are expected to incur a higher amount of medical and caregiving costs than those aged less than 75 years, because they are covered by other types of insurance. Finally, 70% of residents in the SHC group did not answer the question regarding their exercise habits and walking speed. As the analysis in which missing values were imputed showed similar results, we believe that the impact of this limitation on our findings is limited. However, a future consideration is needed whether the missing values were affected by unobserved factors and our imputing process were appropriate.

## Conclusions

We demonstrated a 5-year all-cause cost reduction in residents who participated in the innovative health awareness program, CHAP, and suggested the effect of exercise habits in a rural city in Japan by using real healthcare resource utilization data from individual residents. Our results supported our previous report of the CHAP effect on the future cost reduction and confirmed no long-term effect of SHC suggested by previous reports. individually-based data linkage to external data owned by third parties should be valuable for all local government to improve the health condition of residents.

### Supplementary Information

**Supplementary Material 1.**

## Data Availability

Data cannot be shared publicly because they include personal information. Data of residents in Hirosaki City are available only under the strict agreement between Hirosaki City and Hirosaki University. Data on the CHAP are available from Hirosaki University School of Medicine for researchers who meet the criteria for access to confidential data. Contact information is below: email coi@hirosaki-u.ac.jp.

## Abbreviations

AIC: Akaike’s Information Criterion
CCI: Charlson Comorbidity Index
CHAP: Center of Healthy Aging Program
CHD: Coronary heart disease
SHC: Japan-specific health check-up
