# Supplementary material for: Reduction in all-cause medical and caregiving costs through innovative health awareness projects in a rural area in Japan: a retrospective cohort study
Source: BMC Health Serv Res. 2024 Mar 25;24:370. doi: 10.1186/s12913-024-10836-0 (PMC10962114; doi:10.1186/s12913-024-10836-0)

**S1 Appendix**

**Risk score to estimate the 10-year incidence risk of CHDs**

The detail of this method has been provided by Kinoshita M~~,~~ et al. [17]. The risk score model includes the following variables as risk factors: age, sex, smoking status, HDL-C, LDL-C, blood pressure, impaired glucose tolerance, and a family history of early onset coronary heart disease. Table S1 lists all risk factors and points scored for weighing risks.

**Table S1. Points assigned to CHD risk categories.**

| Risk factor | Category | Points |
| --- | --- | --- |
| Age (years) | 35 to <45 | +30 |
|  | 45 to <55 | +38 |
|  | 55 to <65 | +45 |
|  | 65 to <70 | +51 |
|  | ≥70 | +53 |
| Sex | Female | −7 |
| Current smoking | Yes | +5 |
| HDL-C (mg/dL) | 40 to <60 | −5 |
|  | ≥60 | −6 |
| LDL-C (mg/dL) | 100 to <140 | +5 |
|  | 140 to <160 | +7 |
|  | 160 to <180 | +10 |
|  | ≥180 | +11 |
| Blood pressure (SBP/DBP) (mmHg) | <120 and <80 | −7 |
|  | 140 to <160 and/or 90 to <100 | +4 |
|  | ≥160 and/or ≥100 | +6 |
| Impaired glucose tolerance | Yes | +5 |
| Family history of early onset CHDs | Yes | +5 |

**Table S2. Predicted 10-year risk of CHD by risk score**

| Total score | Predicted risk |
| --- | --- |
| <36 | 0.005 |
| 36 to <41 | 0.01 |
| 41 to <46 | 0.02 |
| 46 to <51 | 0.03 |
| 51 to <56 | 0.05 |
| 56 to <61 | 0.09 |
| 61 to <66 | 0.14 |
| 66 to <71 | 0.22 |
| ≥71 | 0.281† |

* Kinoshita M et al. has reported <0.01 with a median value of 0.005 [17].

†Kinoshita M et al. has reported ≥0.28 with a minimum value of 0.281 [17].

**All-cause medical costs by year of follow-up by subcategories**

**Table S3. All-cause medical costs by year of follow-up by subcategories***

| Subcategory |  | No check-up | SHC | CHAP |
| --- | --- | --- | --- | --- |
| Inpatient | Year 1 | 202,923 | 151,496 | 82,521 |
|  | Year 2 | 209,459 | 150,338 | 66,327 |
|  | Year 3 | 206,148 | 155,124 | 107,711 |
|  | Year 4 | 215,881 | 153,461 | 196,048 |
|  | Year 5 | 214,189 | 168,603 | 125,531 |
| Outpatient | Year 1 | 109,588 | 141,920 | 95,008 |
|  | Year 2 | 108,458 | 140,923 | 97,869 |
|  | Year 3 | 103,924 | 139,234 | 102,581 |
|  | Year 4 | 99,411 | 134,709 | 108,970 |
|  | Year 5 | 106,149 | 136,300 | 122,428 |
| Medication | Year 1 | 316,267 | 444,530 | 306,832 |
|  | Year 2 | 307,725 | 441,632 | 321,060 |
|  | Year 3 | 306,397 | 439,874 | 339,469 |
|  | Year 4 | 301,383 | 433,714 | 344,700 |
|  | Year 5 | 311,293 | 440,690 | 348,712 |

* Adjusted using the length of observable period (unit: year) and calculated for residents who have any costs.

**The distribution of all-cause medical costs**

**Fig S1 The distribution of all-cause medical costs**

**
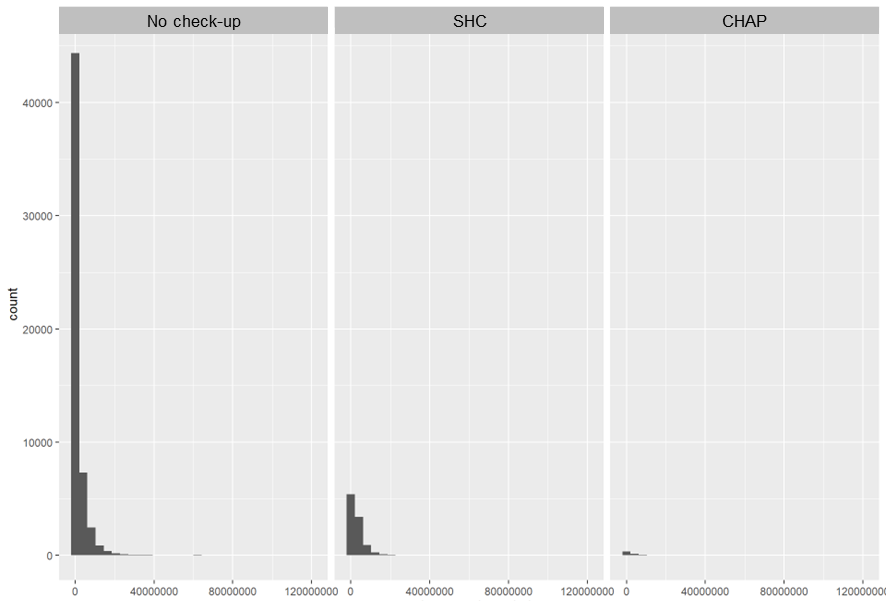
**

**The density plots of the original and imputed datasets**

**Fig S2 The density plots of imputed data and observed data**


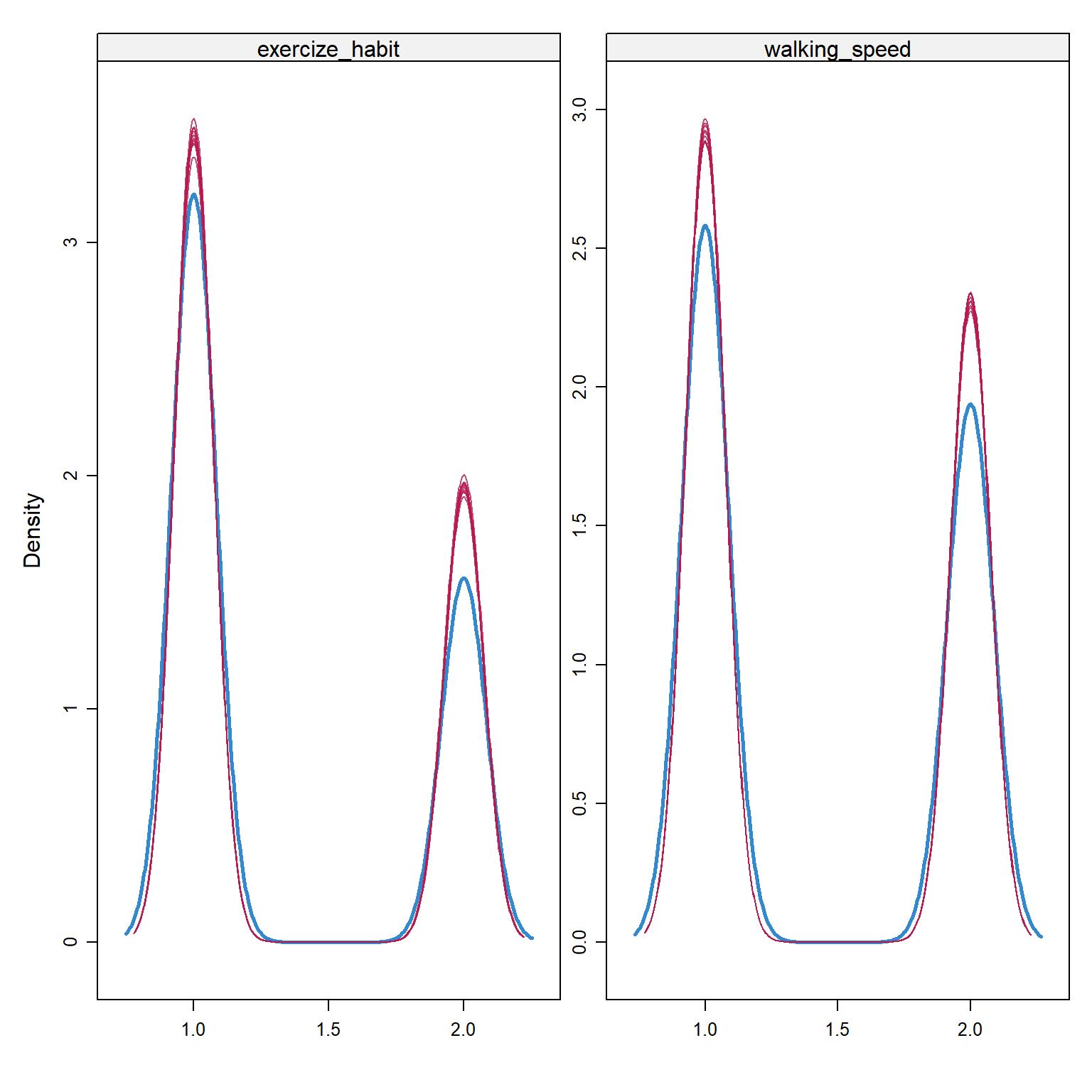

Supplement: Supplementary file 1 — Supplementary Material 1. [file 12913_2024_10836_MOESM1_ESM.docx]
